# Deficits in Agency in Schizophrenia, and Additional Deficits in Body Image, Body Schema, and Internal Timing, in Passivity Symptoms

**DOI:** 10.3389/fpsyt.2014.00126

**Published:** 2014-09-10

**Authors:** Kyran T. Graham, Mathew T. Martin-Iverson, Nicholas P. Holmes, Assen Jablensky, Flavie Waters

**Affiliations:** ^1^Pharmacology, Pharmacy and Anaesthesiology Unit, School of Medicine and Pharmacology, Faculty of Medicine, Dentistry and Health Sciences, The University of Western Australia, Perth, WA, Australia; ^2^Statewide Department of Neurophysiology and Clinical Research Centre, Graylands Hospital, North Metropolitan Health Services – Mental Health, Perth, WA, Australia; ^3^Centre for Integrative Neuroscience and Neurodynamics, School of Psychology and Clinical Language Sciences, University of Reading, Reading, UK; ^4^Centre for Clinical Research in Neuropsychiatry, School of Psychiatry and Clinical Neurosciences, The University of Western Australia, Perth, WA, Australia

**Keywords:** schizophrenia, passivity symptoms, first-rank symptoms, rubber-hand illusion, hand laterality, agency, body schema, body image

## Abstract

Individuals with schizophrenia, particularly those with passivity symptoms, may not feel in control of their actions, believing them to be controlled by external agents. Cognitive operations that contribute to these symptoms may include abnormal processing in agency as well as body representations that deal with body schema and body image. However, these operations in schizophrenia are not fully understood, and the questions of general versus specific deficits in individuals with different symptom profiles remain unanswered. Using the projected-hand illusion (a digital video version of the rubber-hand illusion) with *synchronous* and *asynchronous* stroking (500 ms delay), and a hand laterality judgment task, we assessed sense of agency, body image, and body schema in 53 people with clinically stable schizophrenia (with a current, past, and no history of passivity symptoms) and 48 healthy controls. The results revealed a stable trait in schizophrenia with no difference between clinical subgroups (sense of agency) and some quantitative (specific) differences depending on the passivity symptom profile (body image and body schema). Specifically, a reduced sense of self-agency was a common feature of all clinical subgroups. However, subgroup comparisons showed that individuals with passivity symptoms (both current and past) had significantly greater deficits on tasks assessing body image and body schema, relative to the other groups. In addition, patients with current passivity symptoms failed to demonstrate the normal reduction in body illusion typically seen with a 500 ms delay in visual feedback (*asynchronous* condition), suggesting internal timing problems. Altogether, the results underscore self-abnormalities in schizophrenia, provide evidence for both trait abnormalities and state changes specific to passivity symptoms, and point to a role for internal timing deficits as a mechanistic explanation for external cues becoming a possible source of self-body input.

## Introduction

In the field of cognitive neuroscience, the “sense of self” refers to a complex framework, which is derived from cognitive, sensory, and motor systems. In this context, a subjective experience of “self” is drawn, at least in part, from information gained from body and motor senses. Self-abnormalities in schizophrenia have long been documented in the clinical literature. Kurt Schneider noted that symptoms described “a loss of the very contours of the self” (1), and Bleuler (2) described the tearing apart or splitting of psychic functions. Such self-abnormalities appear to be characteristic of schizophrenia (3, 4), and are particularly pronounced in passivity symptoms (experience of alien control), where individuals do not feel in control of their movements and believe that their actions and intentions are controlled by an external agent. In passivity symptoms, the primary experience is that of a perceptual change regarding how the self is experienced alongside the subjective experience of an external locus of control for internally generated events.

A contemporary model suggests that such abnormalities arise from a failure in the mental operations responsible for predicting the sensory consequences of intended motor commands (the forward model), where the brain “anticipates” an action taking place (5–7). Cognitive self-monitoring models, by contrast, have explained the observed self-distortions as a failure of higher order cognitive processes involving source-monitoring, biases, and *post hoc* inferences that enable coherent self-referencing over time (8, 9). It is becoming clear, however, that these proposals are not adequate or sufficient as theoretical frameworks for motor passivity symptoms (10, 11). Criticisms include that motor commands are neither necessary nor sufficient to engender a sense of agency, and that *post hoc* inferences and biases cannot fully account for pervasive changes in self-experience and self-awareness reported by people with schizophrenia. In support, structured clinical interviews using a clinical–phenomenological approach demonstrate fundamental changes in embodied self-presence, self-experience, and self-judgment in individuals with schizophrenia (12) and in those at high risk of psychosis (13). In addition, disruptions in the forward model should precipitate gross motor problems in people with schizophrenia, for which there is contrary evidence (14, 15).

### Body representation distortions as an alternative framework for explaining self-abnormalities in schizophrenia

A focus on purely motor or cognitive mechanisms fails to consider other somatic and psychological processes that are necessary prerequisites for a coherent sense of self. It was recently suggested that self-deficits in schizophrenia may be better described as broad deficits in body representations that extend beyond self-agency (16). This proposal was drawn from evidence showing that the self emerges from the concurrent activation of multiple body representations, which are derived from multimodal sensory input as well as motor monitoring sources, and that are based on anatomical and neural networks, which play a critical role in one’s sense of self. Body representations are intrinsically linked to one’s sense of awareness, identity, self-concept, and sense of uniqueness. They are needed for the differentiation of body parts and for the accurate performance of purposeful actions.

A general framework for conceptualizing body representations includes at least two important representations: body image and body schema. *Body image* refers to a top-down cognitive representation that integrates the conscious perceptual experiences of one’s body and contributes to one’s belief and attitude about one’s body (17–21). *Body schema* is typically defined as an unconscious dynamic sensory representation that reflects the position and movement of the body and limbs in space (17–20). The validity of these body representations is supported by studies of neurological patients, where localized lesions can selectively impair one or more representations (22–26), and from brain imaging studies pointing to differential activation of neural networks on tasks selective for each body representation (27–31). Finally, for the purposes of the current study, the *sense of agency* is defined as the experience that one is the initiator and in control of one’s actions. The sense of agency is different from body representations as it is critically dependent on actions and intentions (32–34).

### Body representation distortions in people with schizophrenia

As detailed previously, people with schizophrenia have difficulty in correctly attributing agency to self-made movements (35, 36), indicating distortions in agency. There is also emerging evidence for disturbances of these multiple body representations in schizophrenia. For example, empirical findings point to difficulties in imagining movements (37) pointing to deficits in body schema. People with schizophrenia also have abnormal body image, as assessed using a body distortion questionnaire (38). From these findings, it would appear that the internal modeling of the self is weakened or more malleable in people with schizophrenia.

The question of general versus specific deficits in individuals with different symptom profiles, however, has not yet been addressed. Specifically, are these body representation-deficits present in all individuals with schizophrenia or only those with passivity symptoms? According to the philosophical–theoretical tradition of self-disturbances in schizophrenia (3), passivity symptoms represent the more severe and elaborated form of self-disturbances in a continuum from non-psychotic experiences through intermediate phenomena into the manifest psychotic symptoms. Individuals then transit back and forth between manifest psychosis and the intermediary forms as their clinical condition changes over time. According to this view, there should be quantitative differences between people with passivity symptoms compared to individuals with a history of these symptoms and individuals with no lifetime history of passivity. The performance of individuals with schizophrenia with different symptom profile was therefore of interest in the current study.

### Assessing body representations in the current study

Body illusions, such as the rubber-hand illusion, are frequently used to examine processes underlying self-recognition. In the rubber-hand illusion, participants watch a fake hand being stroked, while their own hand is synchronously stroked out of view. This produces an illusory sensation of ownership of the rubber hand and a shift in perceived hand location toward the fake hand. A key requirement of the illusion is that of synchronous input between sensory modalities (tactile and vision). In the asynchronous condition, the illusion can be abolished or diminished by introducing a temporal delay between brush strokes and visual feedback (39). This condition allows an examination of the effects of a timing delay on each type of body representation.

People with schizophrenia tend to experience the rubber-hand illusion more strongly (40, 41) and faster (42) compared to healthy controls. Additionally, the relocation of the perceived position of one’s own hand toward the image (“proprioceptive drift”) has been shown to be greater in schizophrenia than controls, indicating stronger visual capture of proprioceptive information (40). The *projected-*hand illusion, however, has not yet been reported in the schizophrenia literature. The projected-hand illusion uses a live video image of the participant’s own hand projected onto a video screen, allowing a more realistic image of the hand than the traditional “rubber-hand” methodology, more precise control over the timing of brush strokes, as well as enhanced merging of reality into the illusion.

This task assesses two aspects of the sense of self in one experimental set-up. Using a post-performance questionnaire, body image can be assessed on domains of “embodiment (of the ‘other’ projected hand)” and “disembodiment (of one’s own hand),” and the sense of agency with the subjective sensation of motor control (over both the “other” and own hand). Psychometric studies show that illusory sensations over the “other” hand are simultaneously associated with a reduction of the same sensations in the real hand (43). For example, embodiment of the “other” hand is proportionally related to disembodiment of one’s real hand, with the total embodiment of both being equal to one single hand (44, 45). A similar balance also exists with the sense of agency (46). Disembodiment (of limbs) and reduced agency (over actions) are clinical features of persons with passivity symptoms, so performance on such measures are of particular interest.

In order to assess the third type of body representation (body schema), the current study employed the hand laterality task (47). In this task, participants are asked to make a judgment regarding whether an image of a hand is that of a right or left hand by mentally rotating their own hand to match the hand on the screen. Both response times and accuracy are recorded. Evidence that imagined movements are dependent upon the body schema and include findings that performance on this task is influenced by the same biophysical constraints that underlie performed actions (48). A recent study shows that schizophrenia individuals (*n* = 13) were impaired on the task (49), although an analysis of passivity symptoms was not conducted.

### Aims and hypotheses

In the current study, we studied body representations in 53 individuals with schizophrenia and 48 healthy controls on the validated projected-hand illusion (50, 51) and the hand laterality task (47). Individuals with schizophrenia were clustered into subgroups based upon their lifetime history of passivity symptoms. The research questions were as follows: (1) what is the pattern of performance on measures of body schema, body image, and the sense of agency in individuals with schizophrenia compared to controls?; (2) does the evidence point to a stable trait for schizophrenia (no difference between clinical subgroups) or to quantitative differences depending on the passivity symptom profile? Our hypotheses are that body representation distortions will be present in varying degrees in the clinical population: individuals who are currently symptomatic (with passivity symptoms) will have the most severe abnormalities on all body representations, and those with a past history of symptoms, by virtue of their trait vulnerability, will have greater abnormalities than those with no history of symptoms and healthy controls but less than those who are currently symptomatic.

## Materials and Methods

### Participants

The patient sample included individuals with schizophrenia or schizoaffective disorder (53 total, 36 males) recruited from the research database of the WA Family Study of Schizophrenia (52, 53). All patients met both ICD-10 and DSM-IV criteria for a lifetime diagnosis of schizophrenia or schizoaffective disorder, and were community outpatients not currently admitted into a psychiatric hospital and were treated with psychotropic medication. Exclusion criteria included comorbid organic brain disease or substance-use disorder that could account for the psychotic symptoms or language difficulties.

Healthy controls (48 total, 24 males) were recruited through community advertising. Potential controls were excluded if they had a history of a psychotic disorder, or if any of their first-degree relatives had been diagnosed with schizophrenia, schizophrenia-spectrum, or bipolar affective disorder.

The study protocol was explained to all participants and written informed consent was obtained. The study was approved by the North Metropolitan Mental Health Service Human Research Ethics Committee and conformed to the appropriate regulatory standards.

### Clinical evaluation

Clinical evaluation was conducted with the Scales for the Assessment of Positive and Negative Symptoms [SAPS and SANS; (54, 55)]. Passivity symptoms were assessed using the Passivity Symptoms Interview (PSI) (56) with selected items from the Schedule for Clinical Assessment in Neuropsychiatry [SCAN, Version 2.1; items: 17.008, 18.005–18.010, 18.012–18.017, see Ref. (57)]. All symptoms were rated in accordance with stringent definitions and criteria assessed for lifetime history and presence in the last 4 weeks as determined by self-reports and case-note reviews. Patients were rated as having current passivity symptoms (current group) if they reported two or more such symptoms in the past 4 weeks (*n* = 20). Patients were rated as “Past” (*n* = 12) if they had a positive rating of at least two passivity symptoms in the past but not within the past 4 weeks or “Never” (*n* = 21) if they had never experienced these symptoms during any period. Independent classification of patients into groups was conducted by two of the investigators (Kyran T. Graham and Flavie Waters) and rated based on consensus.

### Experimental tasks

#### Hand illusion

**Figure 1:**
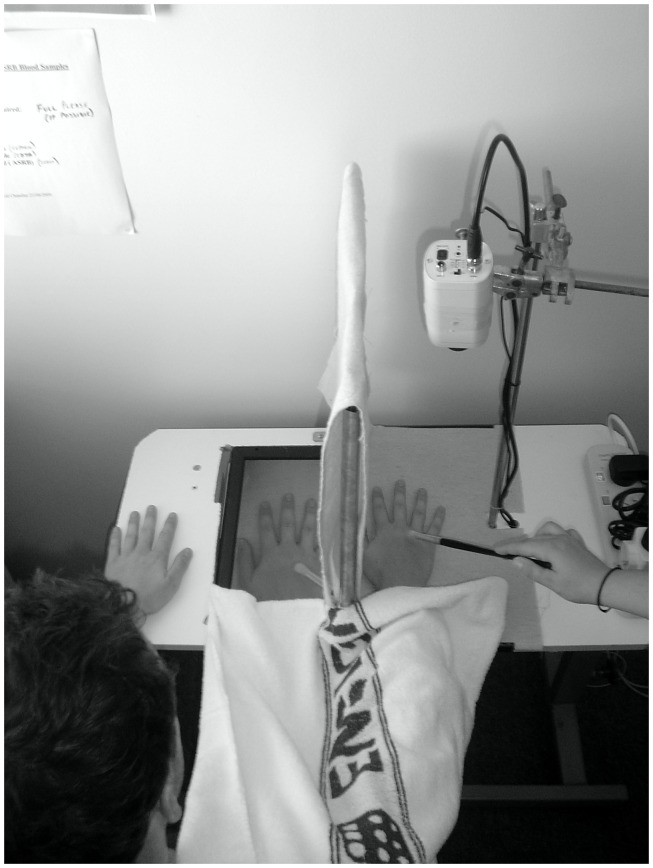
**Photograph of the projected-hand illusion is shown**.

Each participant sat in front of a table with a Fujitsu 17″ color monitor embedded horizontally in the top, with both hands resting on top of the table. The right hand was hidden behind a removable curtain. An image of this hand was captured by an analog camera (AVC-561, AVTECH, Taiwan) and transmitted to the monitor via an analog delay line (DL1B-5379, Ovation Systems Ltd., UK). The real hand and the image of the hand were separated by 15 cm. A photograph of the set-up used can be seen in Figure 1. There were two delay conditions in the illusion; *synchronous* (<10 ms video feedback) and *asynchronous* (an additional imposed 500 ms delay). Participant were exposed to each condition once (3 min each), with the order of presentation being counter-balanced across participants. A 20-item questionnaire assessing the subjective experience of the illusion was administered after each condition (46); adapted from Ref. (43). Items relating to the component Deafference were not included as the component does not pertain to body representations. Each item was rated on a 7-point Likert scale ranging from −3 (strongly disagree) to +3 (strongly agree). A recent PCA (46) identified that the following components could be extracted from the questionnaire, assessing body image (“Disembodiment of own hand” and “Embodiment of the ‘Other’ hand”), and the sense of agency (“Agency over the ‘Other’ hand,” and “Loss of agency over own hand”) in both *synchronous* and *asynchronous* conditions. Table 1 shows the 20 items (Embodiment items 1–8, Disembodiment 13–17, Agency 9–10, and Loss of agency 11–12).

**Table 1:** **Questionnaire items used during the projected-hand illusion**.

It seemed like …	Component
… I was looking directly at my own hand, rather than at an image	Embodiment
… the image began to resemble my real hand	Embodiment
… the image of the hand belonged to me	Embodiment
… the image was my hand	Embodiment
… the image was part of my body	Embodiment
… my hand was in the location where the image was	Embodiment
… the image was in the location where my hand was	Embodiment
… the touch I felt was caused by the paintbrush touching the image	Embodiment
… I could have moved the image of the hand	Agency over the image
… like I was in control of the image	Agency over the image
… I was unable to move my hand	Loss of agency over own hand
… I couldn’t have moved my hand if I had wanted	Loss of agency over own hand
… I couldn’t really tell where my hand was	Disembodiment
… my hand had disappeared	Disembodiment
… my hand was out of my control	Disembodiment
… my hand was moving toward the image	Disembodiment
… the image was moving toward my hand	Disembodiment

#### Hand laterality task

For each trial, a picture of a hand, palm down, was displayed on a computer screen (47). Participants were instructed to indicate if the hand was a left or right hand by pressing an appropriate key on a keyboard. Each picture was either a left or right hand and rotated by either 0°, 90° medially, 90° laterally, or 180°. There were six repeats of each hand/rotation combination for a total of 48 trials per participant. The stimuli were presented in a random order. Participants were instructed not to make major movements of their hands or heads while making the judgments. Four practice trials with feedback were given to each participant before commencing the main experiment. The experiment was produced using E-Prime 1.2 software (Psychology Software Tools, Pittsburgh, PA, USA). In order to rule out possible abnormalities in mental rotation, a similar task was conducted in which the letter F was displayed instead of a hand. The letter was either oriented normally or mirrored along the vertical axis. The same number of trials of letter and rotation combinations was used as the hand laterality task. For both tasks, accuracy and response time were recorded.

### Cognitive tasks

The Wechsler Test of Adult Reading (WTAR) (58) was included as a measure of pre-morbid intelligence. Trail Making Test Form A (TMTA) (59) provided a measure of speed of processing. The Digit Span (DS) provided a measure of attention span (forward span) and working memory (backward span) (59).

### Statistical methods

All statistical analyses and figures were completed using the statistical software R [version 3.0.1; Ref. (60)], and the packages “nlme” (61) and “car” (62). Analyses were performed using linear mixed-effects models with the mean score on the relevant subscale as the dependent variable, delay condition (*synchronous* or *asynchronous*) was the within-subjects variable, group (Controls, Current, Past, or Never) as the between-subjects variable and participant as the random effects term. Similarly, for the hand laterality task, separate models were created for (a) mean accuracy (% incorrect) and (b) mean response time (seconds). For these, group was the between-subjects independent variable and rotation (0°, 90° medial, 90° lateral, and 180°) was the within-subjects variable. Performance (% incorrect and response time) on each rotation for the letter rotation task was included as a covariate in these analyses. Where analysis of deviance (ANODEV) on the terms of the model revealed significant differences, interaction contrasts comparing difference in scores on each of the levels of the factor were performed, i.e., [Controls(Synch) − Controls(Asynch)] − [Current(Synch) − Current (Asynch)]. Alpha was set to 0.05.

## Results

### Projected-hand illusion

Demographic information for participants can be seen in Table 2. Where there were differences between groups, these data were then entered into the projected-hand illusion analyses as covariates. However, there were no significant effects of any of the covariates for the PHI data (*p* > 0.1) and so these were removed from the final model.

**Table 2:** **Demographic information of participants**.

	Controls (*n* = 48)	Never (*n* = 21)	Past (*n* = 12)	Current (*n* = 20)
Sex (M/F)^a^	24/24	14/7	10/2	12/8
Age (years)^b^	46.2 ± 1.68	42.5 ± 1.57	43.6 ± 2.84	44.0 ± 2.06
Years of education^b^	13.7 ± 0.35	12.9 ± 0.37	13.0 ± 0.54	13.7 ± 0.57
WTAR^b^	104 ± 1.9	100 ± 3.3	95 ± 3.4	96 ± 3.2*
Trail Making Test A^b^	31.9 ± 2.82	53.0 ± 7.47***	51.2 ± 11.5***	45.7 ± 8.68**
SAPS composite^b^	–	12.0 ± 2.3^∧∧∧^	19.2 ± 3.5	29.2 ± 3.2
SANS composite^b^	–	21.8 ± 3.6	29.8 ± 4.7	24.7 ± 2.5
Chlorpromazine equivalents (mg)^b^	–	677 ± 121	805 ± 140	754 ± 106

*Mean ± SEM of selected covariates*.

*^a^Fisher’s Exact Test*.

*^b^One-way ANOVA with Tukey’s HSD *post hoc* comparisons (Bonferroni corrected)*.

Different from controls: *p < 0.05, **p < 0.01, ***p < 0.001.

*Different from Pass. Current: ^∧^*p* < 0.05, ^∧∧^*p* < 0.01, ^∧∧∧^*p* < 0.001*.

*Antipsychotic doses converted into chlorpromazine equivalents using the formulae given in (69–71)*.

#### Schizophrenia groups combined

Performance was first examined with a comparison of people with schizophrenia as a group versus healthy controls to determine overall effects of diagnosis while maximizing power to detect an effect. SAPS and SANS scores and chlorpromazine equivalents were included as further covariates in all projected-hand illusion analyses but were removed from the final model, as none were significant. People with schizophrenia reported increased feelings of disembodiment [*F*(1, 99) = 29.5, *p* < 0.0001], and a greater loss of agency over their own hand [*F*(1, 99) = 21.3, *p* < 0.0001] compared to controls, showing greater deficits identifying the experience of their own body.

There were no main effects of group [*F*(1, 99) = 1.83, *p* = 0.18] or interaction [*F*(1, 1498) = 2.65, *p* = 0.10] on the embodiment of the “other” hand component [*F*(1, 97) = 3.63, *p* = 0.06]. Further, there was no significant difference between groups in the sense of agency over the “other” hand [*F*(1, 99) = 0.19, *p* = 0.66].

#### Group comparisons – body image (embodiment of image)

**Figure 2:**
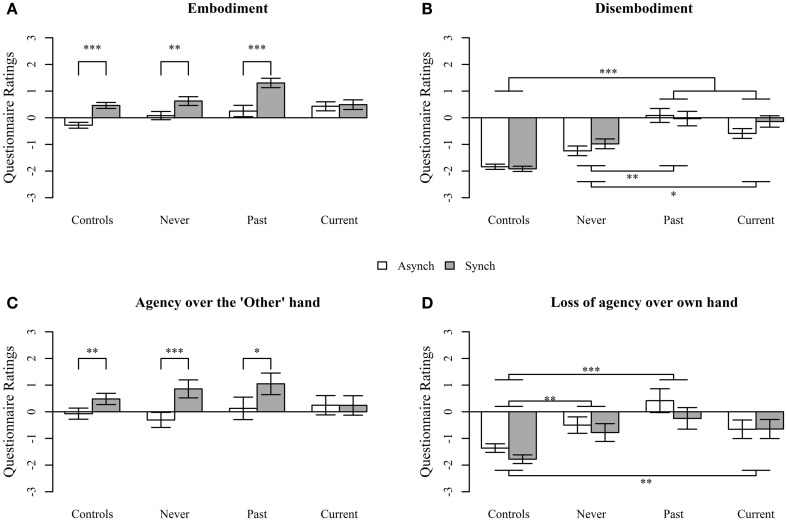
**Questionnaire responses assessing (A) Embodiment, (B) Disembodiment, (C) Agency over the “Other” hand, and (D) Loss of agency over own hand, during the projected-hand illusion after asynchronous (Asynch) and synchronous (Synch) stimulation in controls, people with schizophrenia with no history of passivity symptoms (Never), people with a past history of passivity symptoms (Past), and people with current experiences of passivity symptoms (Current)**. Questions were answered on a 7-point Likert scale. Data are mean ± SEM. *p < 0.05, **p < 0.001, ***p < 0.0001.

Analysis of deviance revealed no main effects of group on Embodiment [*F*(3, 97) = 0.83, *p* = 0.48], but there was a significant main effect of delay [*F*(1, 1496) = 57.8, *p* < 0.0001], with ratings being higher in the *synchronous* condition. There was a significant interaction between group (Controls, Current, Past, and Never) and delay condition [*F*(3, 1496) = 4.94, *p* = 0.002]. Interaction contrasts revealed significant differences between Current and each of the other groups: Controls (*p* = 0.001), Never (*p* = 0.04), and Past (*p* = 0.0006). Controls and patients in the Past and Never groups demonstrated embodiment of the hand in the *synchronou*s condition, which was reduced in the *asynchronous* condition. By contrast, patients in the Current group showed no difference in performance between the *synchronous* and the *asynchronous* conditions, exhibiting embodiment in both conditions (see Figure 2A).

#### Group comparisons – body image (disembodiment of own hand)

For disembodiment (Figure 2B), there was a main effect of group [*F*(3, 97) = 13.1, *p* < 0.0001], but not delay condition [*F*(1, 892) = 1.25, *p* = 0.26] and the interaction was not significant [*F*(3, 892) = 6.78, *p* = 0.08]. Disembodiment of own hand was significantly higher in the Past (*p* < 0.0001), Current (*p* < 0.0001), and the Never groups (*p* = 0.01), relative to controls. The Current and Past groups were marginally significantly different from each other (*p* = 0.05) but both reported higher disembodiment than the Never group (Past *p* = 0.009, Current *p* = 0.04).

#### Group comparisons – agency (agency over the image)

Analysis of deviance revealed no main effect of group [*F*(3, 97) = 0.16, *p* = 0.92], but there was a significant main effect of delay condition [*F*(1, 292) = 19.2, *p* < 0.0001], with an overall increase in reported agency over the “other” hand in the *synchronous* compared to *asynchronous* condition. The interaction between group and delay condition neared, but did not reach, significance [*F*(3, 292) = 7.59, *p* = 0.055]. However, given the *p*-value, it was decided that it was reasonable to perform interaction contrasts. Figure 2C shows that Controls, Never, and Past all demonstrated increased agency over the “other” hand, after *synchronous* compared to *asynchronous* stimulation (treatment contrasts; *p* = 0.007, *p* = 0.002, *p* = 0.02, respectively), while the Current group failed to demonstrate the expected decrease in the *asynchronou*s condition (*p* = 0.98) and reported similar levels of agency after both *synchronous* and *asynchronous* stimulation. However, the only pairwise interaction treatment contrast that was significant was between Current and Never groups (*p* = 0.009).

#### Group comparisons – agency (loss of agency of own hand)

For the loss of agency of own hand component, there was a significant main effect of group [*F*(3, 97) = 25.0, *p* < 0.0001] and a significant effect of delay condition [*F*(1, 293) = 3.97, *p* = 0.046] such that loss of agency ratings were higher in the *synchronous* condition but no significant interaction [*F*(3, 293) = 0.49, *p* = 0.69]. Controls reported significantly less loss of agency over their own hand relative to the Current (*p* = 0.004), Past (*p* < 0.0001), and Never groups (*p* = 0.003), but there were no significant differences between schizophrenia groups (all *p* > 0.1; see Figure 2D).

### Hand laterality task

Scales for the Assessment of Positive Symptoms score, Scales for the Assessment of Negative Symptoms score, and chlorpromazine equivalents were initially included as covariates in all hand laterality analyses, but none had a significant association so they were excluded from the final model.

#### Schizophrenia groups combined (hand laterality task – response time)

As expected, on response time with the schizophrenia groups and healthy controls, the ANODEV displayed a significant main effect of rotation [*F*(3, 700) = 460, *p* < 0.0001] with the response time on 0° trials significantly different from 90° Medial (*p* < 0.0001), 90° Lateral (*p* < 0.0001), and 180° trials (*p* < 0.0001). There was a main effect whereby individuals with schizophrenia had longer response times across all rotations [*F*(1, 99) = 17.7, *p* < 0.0001], as well as an interaction of group and rotation [*F*(3, 693) = 12.8, *p* = 0.005], indicating a further increase in response time on the 90° lateral (*p* < 0.0001) and 180° rotations (*p* < 0.0001) compared to controls.

#### Schizophrenia groups combined (hand laterality task – accuracy)

There were significant positive associations between accuracy on the hand laterality task and WTAR scores [*F*(1, 78) = 15.9, *p* < 0.0001, slope = 0.26], and accuracy on the letter rotation task [*F*(1, 560) = 4.91, *p* < 0.03, slope = 0.06], so these variables were retained as covariates. There was a significant effect of rotation [*F*(3, 560) = 46.2, *p* < 0.0001]. Contrasts demonstrated that accuracy on 0° trials is not different to 90° Medial (*p* = 0.53) or 90° Lateral (*p* = 0.08) trials, but significantly different from 180° trials (*p* < 0.0001). There was no significant main effect of group (schizophrenia group versus Controls) in accuracy [*F*(1, 78) = 0.14, *p* = 0.71]. There was a significant interaction between group and rotation [*F*(3, 560) = 9.13, *p* = 0.03] due to the schizophrenia group being significantly less accurate on the 90° Lateral rotation (*p* = 0.03).

#### Group comparisons (hand laterality task – response times)

**Figure 3:**
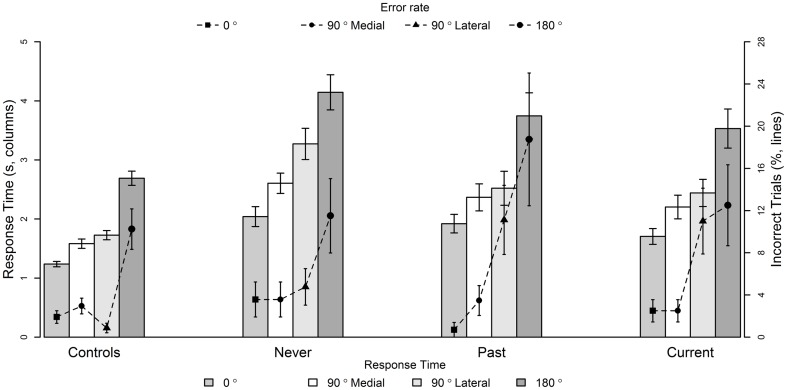
**Mean response times (seconds, columns) and inaccuracy (%, lines) of hand laterality judgments at 0°, 90° Medial, 90° Lateral, and 180° rotations for Controls, people with no history of passivity symptoms (Never), people with a past history of passivity symptoms (Past), and people with current experiences of passivity symptoms (Current)**. Data are mean ± SEM. See text for treatment contrasts.

Response time on the letter rotation task covaried significantly with the response time on the hand rotation task [*F*(1, 687) = 13.9, *p* = 0.0002, slope = 0.10]. However, all significant effects remained so with inclusion of the covariate. No other covariates, including chlorpromazine equivalents, were significant. There was a significant main effect of group on response times [*F*(3, 97) = 20.6, *p* < 0.0001]. There was also a significant interaction between group and rotation type [*F*(9, 687) = 20.9, *p* = 0.01]; response times of Current and Past were significantly longer than controls at 90° lateral (*p* = 0.03 and *p* = 0.03) and 180° rotations (*p* = 0.009 and *p* = 0.005), and Never had significantly greater response times compared to Controls at all rotations (0°, *p* = 0.01; 90° medial, *p* = 0.002; 90° lateral, *p* < 0.0001; 180°, *p* < 0.0001) (Figure 3).

#### Group comparisons (hand laterality task – accuracy)

There was a significant interaction between group and rotation [*F*(9, 554) = 27.9, *p* = 0.001], as well as a significant main effect of rotation [*F*(3, 554) = 47.4, *p* < 0.0001]. To investigate the cause of the interaction between group and rotation type, interaction treatment contrasts were performed. There were no significant group differences at 90° medial rotation (all *p* > 0.3). At 90° lateral rotations, the Current and Past (but not Never) were significantly less accurate than controls (*p* = 0.006 and 0.007, respectively). At 180° rotations, only Past were significantly less accurate than controls at 180° rotations (*p* = 0.0007). There was no main effect of group [*F*(3, 76) = 0.94, *p* = 0.20] on accuracy. In regards to the covariates, higher accuracy on the letter rotation task was associated with higher accuracy of hand laterality judgments [*F*(1, 554) = 4.61, *p* = 0.01, slope = 0.06], and a higher WTAR score was associated with higher accuracy [*F*(1, 76) = 14.9, *p* = 0.002, slope = 0.26]. All significant effects remained after inclusion of the covariates.

## Discussion

The main aim of the current study was to assess the integrity of body representations in individuals with schizophrenia compared to controls and the pattern of performance with regards to the presence of passivity symptoms on a body illusion and a hand laterality task.

### What is the pattern of performance in individuals with schizophrenia compared to controls?

Individuals with schizophrenia showed abnormal performance on both the hand illusion and hand laterality tasks. During the hand illusion, individuals with schizophrenia, as a group, showed increased disembodiment of their own hand, as well as a decreased sense of agency over their own hand, relative to controls.

The hand illusion, with its subjective reports, provides a particularly convenient method to examine components of body representations and self- and non-self-dimensions in one experimental set-up. The current study showed dissociation in performance by people with schizophrenia between *self*-embodiment/agency and *other*-embodiment/agency. Specifically, there was no significant difference between the schizophrenia and controls groups on embodiment and sense of agency over the “other” hand, although the clinical group was particularly impaired on trials requiring the processing of their own (self) body. This perhaps suggests that the representation of other/external people is relatively preserved in schizophrenia, but that the representation of their own body is impaired. In other words, these individuals may be particularly susceptible to disruptions in self-processes, producing a sense of disconnectedness from their own body, but that the embodiment and sense of agency over external objects/bodies are unaffected. In a speculative tone, the imbalance between self- and non-self-representations may give rise to distortions regarding the inference of other people’s intention, perhaps triggering or increasing the vulnerability to delusions.

Disordered self-agency is a common finding in experimental tasks testing the forward model and cognitive self-monitoring models (36, 63–66). However, few studies have demonstrated disembodiment in schizophrenia. While patients frequently complain of diminished representations of the bodily self (1, 4), depersonalization and feeling of disembodiment (67, 68), and self-referential processing difficulties (66, 67), such subjective reports are rarely assessed in experimental conditions. Altogether, the current findings, using a hand illusion, provide support for anomalies in self-agency and self-ownership in this group.

Performance on the hand laterality task provided evidence of additional changes in body schema. On this task, individuals with schizophrenia took significantly longer to respond than controls. In addition, this clinical group had significantly lower accuracy on 90° lateral and 180° rotation trials. These are the most difficult trials, even in healthy groups, and performance is typically less accurate and slower than on the other trials (47). In individuals with schizophrenia, this pattern of performance on error and latency measures could not be explained simply in terms of impaired visuospatial abilities or generally slower responses, since controlling for performance on the letter rotation task with the same rotation conditions did not change the results. Given that the hand laterality task is under the same biophysical constraints as performed actions, the current results point to specific difficulties in the processes involving the synchronization of proprioceptive and tactile inputs into a representation of the body in space in schizophrenia. These findings on the hand laterality task underscore those of de Vignemont et al. (*n* = 13) (49). In contrast to the current study, however, they showed an increase in errors on all rotations in their schizophrenia group relative to controls. Their task was similar to ours, so it is likely that differences in patient characteristics or in statistical power contributed to this small difference in performance.

Together, the current findings point to deficits in sense of agency, body image, and body schema in schizophrenia. Performance on these tasks was not related to chlorpromazine equivalents, so antipsychotics dosages are an unlikely contributor to performance. Similarly, performance on the task was not correlated with other clinical or cognitive performance score. We believe that it is the first report of deficits in multiple body representations in schizophrenia.

### Does the evidence point to a stable trait for schizophrenia (no difference between clinical subgroups) or to quantitative differences depending on the passivity symptom profile?

If abnormal body representations represent a stable trait for schizophrenia *in toto*, then no significant differences among Current (current presence of passivity), Past (past history of passivity), and Never (no history of passivity) would be expected, although they would still perform differently from controls. Only partial evidence was found for this suggestion. Specifically, evidence for such a “stable trait” was only observed in the domain of agency, where a reduced sense of agency over one’s own hand was a common feature of all three patient groups.

By contrast, performance on the other variables supported our initial hypothesis that there should be quantitative differences between people with passivity symptoms (“Current”) compared to individuals with a history of these symptoms (“Past”), and individuals with no lifetime history of passivity (“Never”). Performance on tasks assessing body image suggested quantitative differences depending on the passivity symptom profile of the clinical group. Individuals with passivity symptoms (both current and past) had significantly greater changes in body image as indicated by their higher rating of items relating to disembodiment compared to the group with no history of these symptoms, who in turn reported more disembodiment compared to healthy controls.

In accordance with the above, on the hand laterality task, the Current and Past groups demonstrated reduced accuracy on judgments of the 90° lateral and 180°(Past only) rotations. This finding is in line with demonstrations of impaired performance on a task of motor imagery in people with motor passivity symptoms (37). While this points to problems in body schema, it is important to note that actions and proprioception remain largely unimpaired in this group (14, 15). This suggests that only some subcomponents of body schema are impaired, either in the access pathways to this information or in the integration with other body representations (37).

In sum, the evidence points to both general (trait) deficits in all individuals with schizophrenia (the sense of agency) and quantitative (specific) differences depending on the passivity symptom profile (body image and body schema). Questions remain, however, regarding the processes that separate individuals with current passivity symptoms from those with a history of these symptoms. Both groups show deficits in sense of agency, body image, and body schema, so these processes are not sufficient alone for passivity symptoms. What determines whether patients experience these symptoms? A clue lies in the examination of performance on the hand illusion, specifically on the asynchronous condition.

### Decreased sensitivity to timing delays associated with passivity symptoms

On all measures of the hand illusion involving timing delays, individuals in the Current passivity group distinguished themselves from the other groups. Most remarkably, they failed to demonstrate the normal reduction in the body illusion typically seen with a 500 ms delay in visual feedback (*asynchronous* condition). This performance was specific to those in the Current group, as the other clinical groups (including the Past group) showed the expected illusory decrease on the *asynchronous* condition. In other words, individuals with passivity symptoms continued to experience illusions of embodiment and sense of agency over the “other” hand, when the other groups did not. This suggests that the temporal window that provides links between self and external stimuli is significantly, and abnormally, elongated in people with passivity symptoms. Alternatively, it is possible that the Current group uses temporal cues during multisensory integration to a lesser extent than the other groups.

The functional significance of this finding cannot be understated, given that internal timing precision is critical for a range of processes including sensory–motor awareness and self-recognition (66, 72, 73). Precise timing is needed for the synchronization of motor, cognitive, and sensory signals. It is also needed to shape sensory awareness and in the formation of causal mental associations. Specifically, voluntary actions, which are followed by a sensory event, are perceived as shifted closer together in time than they actually are, a psychological phenomenon termed intentional binding (74), which contributes toward the sense of self-agency. Abnormal internal timing mechanisms in people with passivity symptom therefore have much explanatory power for their disordered self-attribution system. Other evidence is provided by studies showing time perception impairments in individuals with schizophrenia (75, 76). Passivity symptoms studies also show dysfunctions in cognitive and motor timing. Specifically, these individuals perceive external events to be closer in time together than they are (66, 77, 78), which may impact on the integrity of self- and non-self-attribution processes.

The current hand illusion findings are particularly pertinent, because they show that individual with passivity symptoms experiences an illusory sensation of ownership and agency over an image that is spatially and temporally disjointed from the sensorimotor processes linked to their real hand. It is therefore not surprising that these individuals do not feel in control of their movements, and that they experience confusion regarding the origins of their actions and intentions. Such fragmented phenomena would lead to substantial confusion for internally generated events. If a larger window of integration was indeed closely associated with passivity symptoms, it would be expected to have impact on other behaviors and also other non-body-related illusions such as the ventriloquist illusion.

A possible mechanism might occur via dopaminergic pathways. Using an amphetamine challenge in healthy volunteers as a model of psychoses-related responses in the rubber-hand illusion, our group (44) found that amphetamine appeared to increase the temporal envelope of associability of the rubber-hand visual cues to the feel of the stroking (i.e., had a selective effect of increasing the illusion in the *asynchronous* condition) in a profile of performance, which was similar to the pattern of performance in the Current group. Together with their functional role of assigning salience to external stimuli (35), dopaminergic pathways may well contribute to confusion, and misattribution, of agency via changes in the normal temporal window for associability such that external cues become a possible source of body input.

### Strengths and limitations of the current study

It should be noted here that the hand illusion offers significant advantage over other paradigms assessing sense of agency in schizophrenia (35, 36). Notably, subjective reports of *online and prospective* actions (e.g., “I am able to move it”) in the hand illusion are superior to tasks assessing actions *retrospectively* (“I moved it”), therefore overcoming criticisms about the involvement of other cognitive processes (35), which render such retrospective predictions unreliable (36) [also see Ref. (14, 79)]. Such differentiation between prospective and retrospective assessments is thought to be significant when assessing agency reliably (35). That the items of the questionnaire of the current study assessed *prospective* agency possibly explains why there was no significant difference between the schizophrenia groups on the loss of agency over own hand questions; it would appear that the changes in agency are limited to *retrospective* agency in passivity symptoms. Confirmation of this finding could not be carried out as the current study did not assess *retrospective* agency.

A further limitation of the current study is that Current group had a significantly higher level of positive symptoms as assessed on the SAPS. It may therefore be that overall illness severity contributed to the current results, rather than the presence of passivity symptoms. However, several lines of evidence argue against this proposal: (i) there were no significant associations of SAPS scores with any of the dependent variables; (ii) SANS scores did not differ between groups; (iii) chlorpromazine equivalents did not differ between groups; and (iv) the groups did not differ from each other on cognitive performance.

## Conclusion

To conclude, the current study demonstrated both stable traits in schizophrenia (sense of agency) and some quantitative differences depending on passivity symptom profile (body image and body schema). In addition, the presence of passivity symptoms was linked to an enduring experience of body illusion that was resistant to both spatial separation and temporal delay. Our proposal is that passivity symptoms are linked to deficits in body representations encompassing body image and body schema, changes in the sense of agency, alongside internal timing problems that contribute to excessive associability with external sensory stimuli, producing the sensation that one’s actions are controlled by an external agent.

## Author Contributions

Kyran T. Graham conducted all participant testing, statistical analyses and wrote the first draft of the manuscript. Flavie Waters and Assen Jablenksy contributed to the conception of the project and to the design of the study. Kyran T. Graham, Mathew T. Martin-Iverson, and Nicholas P. Holmes provided input into the experimental procedures and testing. All authors contributed to manuscript drafts.

## Conflict of Interest Statement

The authors declare that the research was conducted in the absence of any commercial or financial relationships that could be construed as a potential conflict of interest.
